# Could we adopt serum Tenascin-C assays to determine prognosis in aortic aneurysms and dissections?

**DOI:** 10.1590/1677-5449.200165

**Published:** 2021-08-13

**Authors:** Larissa Cristina França Santos, Mylenne Alinne Falcão de Paiva, Matheus Valois Lapa Santana, Rodrigo Mendes, Pedro Pereira Tenório

**Affiliations:** 1 Universidade Federal do Vale do São Francisco – UNIVASF, Paulo Afonso, BA, Brasil.; 2 Universidade Federal de São Paulo – UNIFESP, São Paulo, SP, Brasil.

**Keywords:** aorta, assay, titers, biomarkers, injury, remodeling

## Abstract

Abdominal aortic aneurysm is an abnormal dilatation, while acute aortic dissection is a delamination of the tunica media, forming a false lumen. Tenascin-C is a glycoprotein that can be found in situations involving tissue damage. The objective of this article is to evaluate whether Tenascin-C assays could be of use for predicting prognosis in abdominal aortic aneurysms and acute aortic dissection. We conducted an integrative literature review, for which four articles were considered eligible. Two of these studies associated higher Tenascin-C levels with protective factors and lower risk of injury, whereas the other two correlated them with worse prognosis. Some authors believe that Tenascin-C could be a candidate biomarker, but these studies are still inconclusive with regard to its role in the clinical outcomes of patients with aneurysms.

## INTRODUCTION

The aorta is an elastic artery that carries blood to the medium-sized distribution arteries. It comprises three tunicae: the intima, made up of endothelium and a connective tissue layer; the media, which has fenestrated elastin layers, fine elastic fibers, and collagen fibers; and the adventitia, which contains collagen fibers, elastic fibers, fibroblasts, macrophages, mast cells, nerve bundles, and lymph vessels.^1^ Changes to the normal architecture of the aorta are related to development of clinical conditions such as abdominal aortic aneurysm (AAA) and acute aortic dissection (AAD).^1^^,^^2^

An aneurysm is an abnormal dilatation, located in blood vessels or the heart and involving all three tunicae. In the aorta, an AAA constitutes an approximately 50% increase in the diameter of the vessel compared with the proximal segment.^3^^,^^4^ AAAs cause elevated mortality when ruptured and a number of different complications if left untreated, such as ruptures and dissections,^5^ but elective surgical repair is associate with low lethality. In parallel, an AAD occurs when there is a separation or tear involving the tunica media, forming a blood-filled channel within the artery wall, which is not necessarily linked to prior dilatation of the vessel. This is a clinically important disease because when AADs rupture, they can hemorrhage into adjacent spaces and manifest acutely.^6^

**Figure 1:**
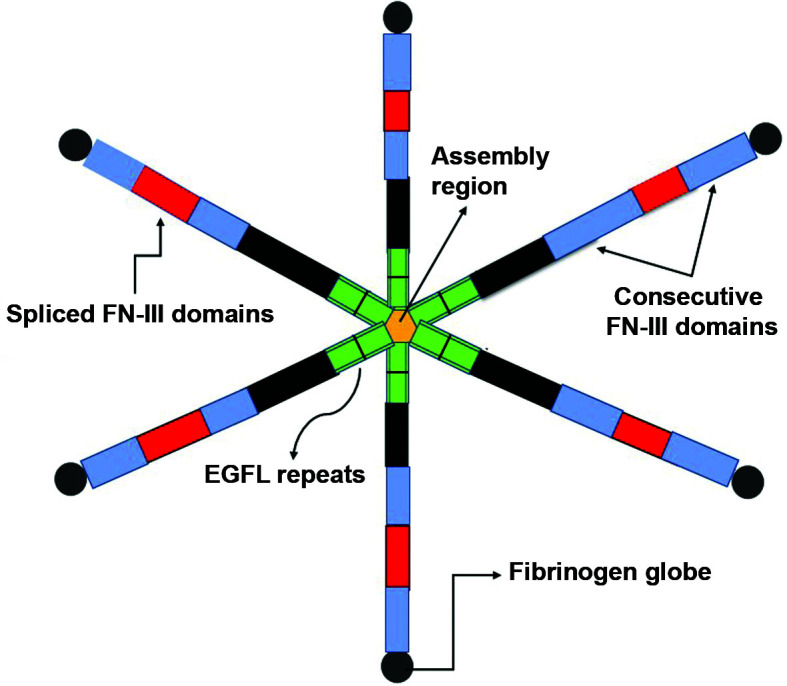
Structure of Tenascin-C. A hexameric glycoprotein composed of individual polypeptides with molecular weights ranging from 180 to 300 kDa. Members of the tenascin family share a similar structure which includes hepta-repeats, epidermal growth factor-like (EGFL) repeats, and fibronectin type III (FNIII) domains.

When destruction of the extracellular matrix (ECM) occurs in the tunica media of the aorta, smooth muscle cells and fibroblasts synthesize Tenascin-C (TN-C), a glycoprotein that is present in responses to inflammatory cytokines (Figure 1) and which can influence cell behavior when it bonds to cell surface receptors or to other matrix proteins.^7^^,^^8^ This glycoprotein has effects on cell adhesion, differentiation, growth control, and apoptosis, and its expression is robustly associated with the embryonic period. In healthy adult tissues its activity is highly regulated and it is related to proinflammatory effects and tissue repair.^9^^,^^10^

In pathological conditions, it is believed that TN-C plays an important role in pathophysiology, as a transducer of signals for tissue repair and as a vascular wall protector through modulation of inflammatory and fibrotic responses in cardiovascular diseases, including AAA and AAD.^11^^,^^12^ Several articles report that serum TN-C levels are elevated in patients with aortic aneurysms and dissections and argue that the protein has potential for use as a biomarker for diagnosis and also as a predictor of outcomes.^13^

Therefore, the objective of this review was to survey the literature with a view to evaluating whether TN-C could be of use in assessing prognosis of AAA and AAD.

## METHOD

This study is an integrative literature review that included articles published in Portuguese and English. The databases searched were PubMed and Science Direct. Preparatory to searching the databases, appropriate Health Sciences Descriptors (Descritores em Ciências da Saúde - DeCS/Bireme) and Medical Subject Headings (MeSH/NCBI-PubMed) were selected, obtaining the following keywords in English: “tenascin”, “aortic dissection” and “aortic aneurysm”. A search strategy with sensitivity and specificity was then employed, using combinations of MeSH terms and direct identification of words used in titles and abstracts. The terms were assembled into search strings using the Boolean operators OR (for synonyms) and AND (for grouping), as follows: (“aortic dissection”[MeSH Terms] OR “aortic dissection”[Title/Abstract]) AND (“tenascin”[MeSH Terms] OR “tenascin”[Title/Abstract]) and (“aortic aneurysm”[MeSH Terms] OR “aortic aneurysm”[Title/Abstract]) AND (“tenascin”[MeSH Terms] OR “tenascin”[Title/Abstract]).

These searches returned 27 articles, and were followed by reading of titles, abstracts, and keywords. After this procedure, articles were ruled eligible if they dealt with TN-C and its relationship with the prognosis of research subjects diagnosed with AAD and AAA or subjects induced to develop these diseases, as shown in Table 1. Once the articles that best matched the subject of the review had been identified, the full texts of four articles were read and the data they reported were classified using the Grading of Recommendations Assessment, Development and Evaluation (GRADE) system .

**Table 1:** Summary of the main findings identified in articles that conducted studies on human beings.

**Authors**	**Studies**	**Study design**	**Results**	**Evidence level**
Nozato et al.^14^	Impact of serum Tenascin-C on the aortic healing process during the chronic stage of type B acute aortic dissection	Experiment based on blood sampled on the seventh day after hospital admission from 26 patients admitted to a hospital in Japan	Elevated Tenascin-C levels in patients may predict regression of acute aortic dissection and induce a protective effect in the chronic stage of aortic dissection	2b
Guo et al.^7^	The role of serum Tenascin-C in predicting in-hospital death in acute aortic dissection	Analysis of the outcomes of 109 patients admitted to a hospital in China who were diagnosed with acute aortic dissection	Elevated serum Tenascin-C levels were associated with a higher probability of death in patients with acute aortic dissection.	2b

## DISCUSSION

### Serum TN-C analysis

The reference values analyzed to evaluate patient prognosis varied depending on the methodology employed. Nozato et al. defined a serum level greater than or equal to 81.8 ng/mL as the cutoff for predicting regression of AAD.^14^ The study sample comprised 26 patients admitted to hospital and diagnosed with type B AAD, which only involves the descending aorta. In this study, blood samples were drawn and serum TN-C levels were analyzed on the seventh day after hospital admission.^14^ Later, a regression analysis was conducted, by which the authors identified that elevated serum TN-C levels could be indicative of a low probability of widening of the aorta as the disease progresses to the chronic state. They found a negative correlation between serum TN-C levels and change in aorta diameter. Based on this negative correlation, they concluded that elevated serum TN-C levels on the seventh day after hospital admission may not merely be predictive of AAD regression, but could actually induce a protective effect against expansion of the aortic injury during development of the chronic stage of the disease.^14^

Nozato et al. also reported that serum TN-C levels reached a mean of 103.4±47.9 ng/mL in the group of patients in whom AAD receded. They argue that, although it is still unclear how the events that lead to AAD are triggered, it is known that formation of a false lumen provokes an inflammatory response and then a reparative process is initiated later.^14^ TN-C levels are elevated during the inflammatory and reparative processes and this is associated with several functions, such as modulation of the inflammatory process or tissue repair and even fibrosis. In order to support their hypothesis, the authors cited study findings providing evidence that in the absence TN-C, there is insufficient production of ECM proteins and the inflammatory process is exacerbated.^14^ This information fits with the hypothesis of the importance of TN-C for adequate remodeling of the aorta and protection against new episodes of AAD in the future.

Iamanaka-Yoshida and Matsumoto did not mention reference values.^12^ They too emphasized the role played by Tenascin-X (TN-X) in AAD and AAA, but did not go into details about the levels associated with prognosis in each disease or explain whether there is a relationship between TN-C and TN-X.^12^ They recommend that analysis of serum TN-C levels should be performed at the time of hospital admission and 7 days later.^12^

Iamanaka-Yoshida and Matsumoto demonstrated in their article that when TN-C is induced by destructive mechanical stress, such as an over-activated renin-angiotensin-aldosterone system, it can protect tissues by helping to reduce an inflammatory reaction provoked by the tissue damage.^12^ In an experiment with mice, Iamanaka-Yoshida and Matsumoto observed that tumor necrosis factor (TNFα) had triggered a proinflammatory response while simultaneously suppressing expression of genes linked to collagen production.^12^ *In vitro*, it was observed that exogenous TN-C suppressed proinflammatory gene expression in smooth muscle cells of the thoracic aorta, acting in the opposite manner to TNFα. Production of TN-C enabled expression of the genes responsible for collagen production, making the repair process possible. These authors therefore arrived at the conclusion that TN-C provoked synthesis of ECM as a reparative response to the tissue damage and suppressed inflammatory responses of vascular smooth muscle cells specifically in the case of AAD.

However, Iamanaka-Yoshida and Matsumoto also believe that the elevated TN-C levels identified during inflammatory responses, in the case of AAD, constitute a response that is necessary for repair of tissue architecture.^12^ In this sense, their perspective is that TN-C may be primarily related to fine tuning of inflammatory reactions during tissue injury and repair.

Schaefer et al. reported figures valid for mice: in a group of knockout mice (KO), a mean level of 1.39±0.25 μ/mL was observed and in a group of wildtype mice (WT) the mean level was 1.67±0.22 μg/mL.^15^ The value observed in the control KO group was 0.92±0.08 and the value in the control WT group was 0.96±0.22.^15^ They investigated the relationship between TN-C deficiency and its capacity to attenuate AAA formation.^15^ They conducted laparotomies on male mice and then, in some of the mice from the KO group, in which the genes responsible for transcription of TN-C had been knocked out, induced AAA by periaortic administration of calcium chloride 0.5 M for 15 minutes, while in some of the mice in the WT group they conducted the same procedure, but incubated the artery in saline solution. The authors measured the diameter of the abdominal aorta before aneurysm induction and three and ten weeks after aortic transplant, calculating the proportions between the measurements.^15^

The main findings indicated that in the induced aneurysm groups, the proportion of aorta diameters was smaller in KO mice than in the control group, at both the third and tenth weeks. It was also found that elastin rupture in the tunica media was significantly smaller in KO mice than in the control group 10 weeks after aneurysm induction. Additionally, significant changes to the diameter of the artery were not observed in the control group. Based on these results, the authors associated TN-C deficiency with a lower probability of AAA formation.^15^

Guo et al. identified that patients who survived AAD had serum TN-C levels in the range from 58.3 to 99.3 pg/mL, whereas the levels in patients who did not survive ranged from 112.4 to 163.4 pg/mL.^9^ They also pointed out that using a combination of TN-C and D-dimer assays increased the capacity to predict mortality among hospitalized patients, achieving sensitivity of 90.3% and specificity of 88.4%.^9^ Guo et al. provided more detail than previous studies, specifying collection of 5 mL of venous blood at the time of hospital admission from patients diagnosed with AAD and had manifested symptoms at least 48 hours previously.^9^ Blood samples were collected into pro-coagulation tubes and centrifuged at 3,000 r/minute for 5 minutes. The serum was collected and stored at -80 ºC and analyzed by enzyme-linked immunosorbent assay (ELISA), as shown in Table 2. According to these authors, the proinflammatory action of TN-C prevails, provoking histological destruction of the aorta walls.^9^ These researchers observed higher serum TN-C levels in patients who did not survive than in those who did survive after admission for an acute AAD episode.^9^ It should be noted that one inclusion criterion was that onset of symptoms was at least 14 days earlier. Based on these results, the authors concluded that the increased concentration of TN-C is directly proportional to the severity of the process, indicating that patients with higher levels have a higher probability of death.

**Table 2:** Reference values for Tenascin-C and their respective prognoses. This table only lists articles that studied human beings.

**Authors**	**Reference values**	**Prognosis**
Nozato et al.^14^	81.8 ng/mL	≥ 81.8: regression of acute aortic dissection
Guo et al.^7^	58.3 to 99.3 pg/mL	58.3 to 99.3: possibility of survival of acute aortic dissection
	112.4 to 163.4 pg/mL	112.4 to 163.4: higher probability of death from the same disease

### Limitations of the studies

Nozato et al. pointed out that TN-C is not a good biomarker if the patient has renal dysfunction, because their serum levels will tend to accumulate with no relation to AAD.^14^ For this reason, these authors recommended conducting studies that take additional clinical parameters into account. Iamanaka-Yoshida and Matsumoto noted that TN-C is not an appropriate biomarker if the person has one of the following syndromes: Marfan, Loeys-Dietz, or Ehlers-Danlos. These syndromes are caused by genetic mutations that predispose individuals to development of AAD.^12^ Schaefer et al. conducted their experiment on mice.^15^ In this case, it is clear that studies in humans need to advance in order to investigate the possibility of TN-C acting as a biomarker of AAA.

Guo et al. listed one of the main limitations as the fact that significant fluctuations in serum TN-C levels had been observed between individuals and at different times, which could not be controlled.^9^ They also considered that the TN-C values identified are only valid in the context of hospitalized patients, stressing the need for more studies, because the study was subject to limitations and they were unable to follow-up patients after hospital discharge.

## CONCLUSIONS

The possibility that TN-C could come to be used for evaluating prognosis in AAA and AAD cases in humans was one of the premises adopted in the articles by Guo et al.^7^ and Nozato et al.,^14^ providing that additional clinical investigations are conducted into the subject. While the articles by Iamanaka-Yoshida and Matsumoto^12^ and Schaefer et al.^15^ are based on animal studies, they recognize that future investigations could reveal the role of TN-C in prognosis.

While this important conclusion was observed, practical applications of TN-C are still subject to additional evidence, in view of a series of limitations. More studies must be conducted before this glycoprotein can be used widely. One question that still lacks a definitive answer relates to interpretation of elevated serum TN-C levels. The debate on whether these levels are related to poor prognosis or a possibility of better patient recovery is still ongoing.

Although Guo et al.,^7^ Nozato et al.,^14^ Iamanaka-Yoshida and Matsumoto,^12^ and Schaefer et al.^15^ believe that TN-C could be an eligible biomarker, these studies are still inconclusive with regard to the clinical outcomes of the research subjects. Further research is needed to enable a better understanding of the role of TN-C in the prognosis of these diseases.

In view of the above, there is a clear need for more research before TN-C can be used as a biomarker, considering the scarcity of studies. Moreover, the studies that do exist have small samples and it is therefore difficult to indicate reference values in the absence of validated cohorts.
