# Supplementary material for: Could we adopt serum Tenascin-C assays to determine prognosis in aortic aneurysms and dissections?
Source: J Vasc Bras. 2021 Aug 13;20:e20200165. doi: 10.1590/1677-5449.200165 (PMC8366403; doi:10.1590/1677-5449.200165)
Supplement: Translation

# Translation: Podemos adotar a dosagem sérica da Tenascina-C na avaliação prognóstica do aneurisma e dissecção da aorta?

**Authors:** Larissa Cristina França Santos, Mylenne Alinne Falcão de Paiva, Matheus Valois Lapa Santana, Rodrigo Mendes, Pedro Pereira Tenório

**Affiliations:** 1 Universidade Federal do Vale do São Francisco – UNIVASF, Paulo Afonso, BA, Brasil.; 2 Universidade Federal de São Paulo – UNIFESP, São Paulo, SP, Brasil.

**Keywords:** aorta, dosagem, biomarcadores, lesão, remodelação

## Abstract

O aneurisma da aorta abdominal corresponde a uma dilatação anormal, enquanto a dissecção aórtica aguda é uma delaminação da túnica média com formação de um falso lúmen. A Tenascina-C é uma glicoproteína que pode ser encontrada em situações de lesão tecidual. Nesse sentido, este artigo pretendeu avaliar se a Tenascina-C pode auxiliar na avaliação do prognóstico do aneurisma da aorta abdominal e da dissecção aórtica aguda. Realizou-se uma revisão integrativa da literatura em que foram considerados elegíveis quatro artigos, sendo que dois associaram maiores níveis da Tenascina-C a fatores de proteção e menor risco de lesões, enquanto dois correlacionaram com prognóstico pior. Alguns autores acreditam que a Tenascina-C poderia ser um biomarcador elegível, mas esses estudos ainda são inconclusivos no que diz respeito a seu papel no desfecho clínico dos pacientes com aneurismas.

## INTRODUÇÃO

A aorta é uma artéria elástica que conduz sangue para as artérias de distribuição de tamanho médio. É composta por três túnicas: a íntima, constituída por um endotélio e uma camada de tecido conjuntivo; a média, que apresenta camadas fenestradas de elastina, finas fibras elásticas e de colágeno; e a adventícia, que contém fibras colágenas, elásticas, fibroblastos, macrófagos, mastócitos, feixes nervosos e vasos linfáticos^1^. A alteração da arquitetura normal da aorta está relacionada ao desenvolvimento de condições clínicas como o aneurisma da aorta abdominal (AAA) e a dissecção aórtica aguda (DAA)^1^^,^ 2.

O aneurisma consiste em uma dilatação anormal e localizada em vasos e no coração, envolvendo as três túnicas. Na aorta, o AAA corresponde a um aumento do diâmetro do vaso de aproximadamente 50% em comparação com o segmento proximal^3^^,^ 4. Apresenta alta taxa de mortalidade quando há ruptura, além de apresentar algumas complicações, quando não tratado, como as roturas e as dissecções^5^, mas tem baixa taxa de letalidade com uma correção cirúrgica eletiva. Em paralelo, a DAA ocorre quando há uma separação ou rasgadura da túnica média, formando um canal cheio de sangue dentro da parede da artéria, sem, no entanto, estar necessariamente associada com uma dilatação prévia do vaso. Trata-se de uma doença de importância clínica, pois seu rompimento pode causar hemorragia em espaços adjacentes e manifesta-se agudamente^6^.

Na túnica média da aorta, onde há destruição da matriz extracelular (MEC), células do tecido muscular liso e fibroblastos sintetizam a Tenascina-C (TN-C), uma glicoproteína presente em resposta às citocinas inflamatórias (Figura 1) e que pode influenciar o comportamento celular ao ligar-se a receptores de superfície celular ou a outras proteínas matriciais^7^^,^ 8. Essa glicoproteína tem efeitos sobre adesão celular, diferenciação, controle de crescimento e apoptose, estando sua expressão fortemente relacionada ao período embrionário. No tecido adulto saudável, a sua ação é altamente regulada e está relacionada a efeitos pró-inflamatórios e cicatrização^9^^,^ 10.

**Figura 1:** Estrutura da Tenascina-C. Glicoproteína hexamérica composta por polipeptídios individuais com pesos moleculares que variam de 180 a 300 kDa. O grupo das Tenascinas partilham de um padrão estrutural semelhante que engloba hepta repetições, repetições de fator de crescimento epidérmico (EGFL) e domínios de fibronectina tipo III (FNIII).

Em condições patológicas, acredita-se que a TN-C desempenhe papel importante na fisiopatologia, como transdutores de sinais para reparação tecidual e como protetor da parede vascular ao modular as respostas inflamatórias e fibrosas de doenças cardiovasculares, como AAA e a DAA^11^^,^ 12. Muitos artigos relatam que, nos pacientes com aneurisma e dissecção da aorta, o nível sérico de TN-C encontra-se elevado e defendem que a proteína tem potencial para ser usada como biomarcador para diagnósticos e também como preditor de desfechos^13^.

Portanto, o objetivo desta revisão foi realizar um levantamento na literatura com o propósito de avaliar se a TN-C pode auxiliar na avaliação do prognóstico do AAA e da DAA.

## MÉTODO

O presente trabalho consistiu em uma revisão integrativa da literatura, em que foram incluídos artigos nas línguas portuguesa e inglesa. As bases de dados utilizadas foram a PubMed e a *Science Direct*. Para busca nos bancos de dados, utilizou-se, inicialmente, a estratégia de identificar os Descritores em Ciências da Saúde (DeCS/Bireme) e os *Medical Subject Headings* (MeSH/NCBI-PubMed), e obteve-se os seguintes descritores na língua inglesa: “tenascin”, “aortic dissection” e “aortic aneurysm”. Após isso, foi realizada uma estratégia de busca sensível e específica, utilizando a associação dos termos MeSH e da busca direta pelas palavras em título e resumo. Os descritores foram organizados na busca com os operadores booleanos OR (para sinônimos) e AND (para agrupamento) de forma que as ferramentas de busca obtidas fossem: (“aortic dissection”[MeSH Terms] OR “aortic dissection”[Title/Abstract]) AND (“tenascin”[MeSH Terms] OR “tenascin”[Title/Abstract]) e (“aortic aneurysm”[MeSH Terms] OR “aortic aneurysm”[Title/Abstract]) AND (“tenascin”[MeSH Terms] OR “tenascin”[Title/Abstract]).

A busca obteve 27 artigos, e foi realizada a leitura dos títulos, resumos e palavras-chave. Decorrido esse procedimento, foram considerados elegíveis apenas aqueles que abordavam a TN-C e sua relação com o prognóstico dos sujeitos de pesquisa diagnosticados com DAA e AAA ou que foram induzidos a desenvolver as referidas doenças, conforme a Tabela 1. Uma vez identificados os artigos com maior aderência ao tema, foi feita a leitura completa de quatro artigos, e os dados apresentados por eles foram inseridos no sistema *Grading of Recommendations Assessment, Development and Evaluation* (GRADE).

**Tabela 1:** Esquematização dos principais achados identificados nos artigos cujos ensaios foram realizados em seres humanos.

**Autores**	**Trabalhos**	**Desenho do estudo**	**Resultados**	**Nível de evidência**
Nozato et al.^14^	Impacto dos níveis séricos de Tenascina-C durante o processo reparador do estágio crônico da dissecção aórtica aguda do tipo B	Experimento realizado a partir da coleta sanguínea feita no sétimo dia após admissão hospitalar de 26 pacientes admitidos em um hospital japonês	Níveis elevados de Tenascina-C em pacientes podem predizer regressão da dissecção aórtica aguda e induzir efeito protetor no estágio crônico da dissecção aórtica	2b
Guo et al.^7^	O papel dos níveis séricos de Tenascina-C no prognóstico fatal de pacientes hospitalizados devido à dissecção aórtica aguda	Acompanhamento de 109 pacientes admitidos em hospital chinês diagnosticados com dissecção aórtica aguda	Níveis séricos de Tenascina-C elevados foram associados à maior probabilidade de os pacientes com crise aguda de dissecção aórtica evoluírem para óbito.	2b

## DISCUSSÃO

### Análise sérica da TN-C

Os valores de referência analisados para a avaliação do prognóstico dos pacientes variaram conforme a metodologia empregada. Nozato et al. definiram o nível sérico maior ou igual a 81,8 ng/mL como o valor de referência para predizer regressão da DAA^14^. Os sujeitos de pesquisa foram 26 pacientes admitidos no hospital e diagnosticados com DAA do tipo B, a qual envolve apenas a aorta descendente. Nesse estudo, foi coletada amostra sanguínea e analisados os níveis séricos de TN-C no sétimo dia após admissão hospitalar^14^. Posteriormente, foi realizada uma análise de regressão, por meio da qual os autores identificaram que níveis séricos elevados de TN-C podem ser um indicativo de baixa probabilidade de alargamento da aorta ao longo do processo de cronificação da doença. Dessa forma, constatou-se correlação negativa entre níveis séricos de TN-C e a variação do diâmetro da aorta. A partir dessa correlação negativa, os autores concluíram que níveis séricos elevados de TN-C no sétimo dia após admissão hospitalar pode não apenas predizer a regressão da DAA, como também induzir um efeito protetor em relação ao alargamento da lesão aórtica durante o desenvolvimento do estágio crônico da doença^14^.

Ainda de acordo com Nozato et al., os níveis séricos de TN-C alcançaram uma média de 103,4±47,9 ng/mL no grupo de pacientes em que a DAA regrediu. Os autores argumentaram que, apesar de ainda não se ter clareza acerca de como se encadeiam os eventos na origem da DAA, sabe-se que a formação de um pseudolúmen promove uma resposta inflamatória e, posteriormente, um processo reparador é iniciado^14^. Durante os processos inflamatório e reparador, os níveis de TN-C estão elevados, e isso está associado a várias funções, como modulação do processo inflamatório ou reparação tecidual e ainda fibrose. Para ratificar sua hipótese, os autores expuseram estudos cujos achados evidenciaram que, na ausência de TN-C, ocorria produção insuficiente de proteínas da MEC e o processo inflamatório se exacerbava^14^. Tais informações coadunam com a hipótese levantada sobre a importância da TN-C para o remodelamento adequado da aorta e a proteção contra novos episódios de DAA no futuro.

Iamanaka-Yoshida e Matsumoto não mencionaram valores de referência^12^. Eles também enfatizaram o papel da Tenascina-X (TN-X) frente a DAA e no AAA, mas não se aprofundaram quanto às dosagens referentes ao prognóstico para cada doença nem elucidaram se existe relação entre a TN-C e a TN-X^12^. Segundo eles, a análise dos níveis séricos de TN-C deve ser feita no momento da admissão hospitalar e sete dias depois^12^.

Iamanaka-Yoshida e Matsumoto demonstraram em seu artigo que a TN-C, quando induzida por um estresse mecânico destrutivo, como o sistema renina-angiotensina-aldosterona, fortemente ativado, pode proteger o tecido por meio do auxílio na redução de uma reação inflamatória provocada pela lesão tecidual^12^. Em um experimento com camundongos, Iamanaka-Yoshida e Matsumoto observaram que o fator de necrose tumoral (TNFα) havia desencadeado resposta pró-inflamatória ao mesmo tempo em que suprimiu a expressão de genes ligados à produção de colágeno^12^. *In vitro*, foi relatado que a TN-C exógena suprimiu a expressão gênica pró-inflamatória em células do músculo liso da aorta torácica, atuando de maneira contrária ao TNFα. A produção da TN-C possibilitou a expressão dos genes responsáveis pela produção de colágeno, viabilizando o processo reparador. A conclusão a que chegaram os autores, portanto, foi que a TN-C promoveu a síntese da MEC como uma resposta reparadora à lesão tecidual e suprimiu as respostas inflamatórias das células do músculo liso vascular no caso específico da DAA.

Iamanaka-Yoshida e Matsumoto, entretanto, acreditam que os níveis elevados de TN-C identificados durante as respostas inflamatórias, no caso da DAA, constituem uma resposta necessária para o reparo da arquitetura tecidual^12^. Nesse sentido, a perspectiva, segundo eles, é que a TN-C pode estar principalmente relacionada com o ajuste fino das reações inflamatórias durante a lesão e a restauração do tecido.

Schaefer et al. trouxeram números válidos para camundongos: no grupo de camundongos *knockout* (KO) com aneurisma induzido, foi identificada uma média de 1,39±0,25 μ/mL; no grupo de camundongos selvagens (WT) com aneurisma induzido, de 1,67±0,22 μg/mL^15^. No grupo controle KO, o valor identificado foi de 0,92±0,08; enquanto no grupo controle WT, o valor foi de 0,96±0,22^15^. Schaefer et al. avaliaram a relação entre a deficiência da TN-C e sua capacidade de atenuar a formação do AAA^15^. Para tanto, os autores realizaram laparotomia em camundongos machos. Em seguida, foi induzido o AAA em alguns camundongos do grupo KO para os genes responsáveis pela transcrição da TN-C por meio da aplicação periaórtica de cloreto de cálcio 0,5 M durante 15 minutos. Em alguns camundongos do grupo WT, foi realizado o mesmo procedimento, mas a artéria foi incubada em solução salina. Os autores mensuraram o diâmetro da aorta abdominal antes da indução do aneurisma e nas terceira e décima semanas após o transplante aórtico, calculando a proporção entre as medidas encontradas^15^.

Os principais achados apontaram que, no grupo que teve o aneurisma induzido, a proporção dos diâmetros da aorta era menor nos camundongos KO do que no grupo controle, tanto na terceira quanto na décima semana. Também se identificou que a ruptura da elastina na túnica média era significativamente menor nos camundongos KO do que no grupo controle 10 semanas após a indução do aneurisma. Além disso, no grupo controle, não foram observadas alterações significativas no diâmetro da artéria. De acordo com esses resultados, os autores associaram a deficiência da TN-C com uma menor probabilidade de formação do AAA^15^.

Guo et al. identificaram os níveis séricos de TN-C variando de 58,3 a 99,3 pg/mL nos pacientes que sobreviveram à DAA, enquanto os valores transitaram de 112,4 a 163,4 pg/mL nos pacientes que não sobreviveram^9^. Também destacaram que a combinação da análise da TN-C com o D-dímero aumenta a capacidade preditiva de mortalidade dos pacientes hospitalizados, com sensibilidade de 90,3% e especificidade de 88,4%^9^. Guo et al. foi mais detalhista do que os estudos anteriores, especificando a realização da coleta de 5 mL de sangue venoso no momento da admissão hospitalar dos pacientes diagnosticados com DAA e que manifestavam os sintomas há no máximo 48 horas^9^. A amostra sanguínea foi colocada em tubos pró-coagulação e centrifugadas a 3.000 r/minutos durante cinco minutos. Posteriormente, o soro foi coletado, armazenado a -80 ºC e analisado por meio de ensaio de imunoabsorção enzimática (ELISA), de acordo com a Tabela 2. Segundo eles, a atuação pró-inflamatória da TN-C se sobressai, promovendo a destruição histológica das paredes da aorta^9^. Os pesquisadores observaram níveis séricos mais elevados de TN-C nos pacientes não sobreviventes do que naqueles que sobreviveram após admissão devido ao episódio agudo de DAA^9^. Cabe destacar que o critério de inclusão era que o início dos sintomas tivessem se dado há no máximo 14 dias. Diante desse resultado, os autores concluíram que o aumento da concentração de TN-C é diretamente proporcional à gravidade do processo, indicando que pacientes com níveis mais elevados apresentam maior probabilidade de evoluírem a óbito.

**Tabela 2:** Valores de referência para a Tenascina-C e seus respectivos prognósticos. Constam, nesta tabela, apenas os artigos que realizaram ensaios em seres humanos.

**Autores**	**Valores de referência**	**Prognóstico**
Nozato et al.^14^	81,8 ng/mL	≥ 81,8: regressão de dissecção aguda aórtica
Guo et al.^7^	58,3 a 99,3 pg/mL	58,3 a 99,3: possibilidade de sobreviver à dissecção aórtica aguda
	112,4 a 163,4 pg/mL	112,4 a 163,4: maior probabilidade de óbito para a mesma doença

### Limitações dos estudos

Nozato et al. assinalaram que a TN-C não é um bom biomarcador se o paciente sofrer de disfunção renal, pois seus níveis séricos tendem a se acumular sem guardar qualquer relação com a DAA^14^. Por isso, esses autores recomendaram mais estudos, que levassem em conta outros parâmetros clínicos. Iamanaka-Yoshida e Matsumoto pontuaram que a TN-C não se constitui em um biomarcador adequado quando o indivíduo possui alguma das seguintes síndromes: Marfan, Loeys-Dietz, Ehlers-Danlos. Tais síndromes se caracterizam por mutações genéticas que já predispõem os indivíduos a desenvolverem a DAA^12^. Schaefer et al. realizaram experimento em camundongos^15^. Nesse caso, evidencia-se a necessidade de avançar os estudos em humanos para verificar a possibilidade de a TN-C atuar como biomarcador para a AAA.

Guo et al. apontaram como principais limitações o fato de terem sido observadas flutuações significativas nos níveis séricos de TN-C entre os indivíduos e em marcos temporais diversos, as quais não puderam ser acompanhadas^9^. Além disso, eles consideraram que valores encontrados para a TN-C são válidos apenas no contexto de pacientes hospitalizados, ressaltando a necessidade de mais estudos, pois houve limitações na execução da pesquisa, não havendo condições de acompanhamento dos pacientes após alta hospitalar.

## CONCLUSÃO

A consideração de que a TN-C pode vir a atuar na avaliação do prognóstico frente a AAA e DAA em humanos foi uma premissa adotada nos artigos de Guo et al.^7^ e de Nozato et al.^14^, desde que sejam feitas maiores investigações clínicas nessa área. Os artigos de Iamanaka-Yoshida e Matsumoto^12^ e de Schaefer et al.^15^, apesar de desenvolvidos em animais, reconhecem que investigações futuras podem vir a revelar o papel da TN-C para o prognóstico.

Embora tenha sido observada essa importante constatação, a utilização prática da TN-C ainda carece de mais esclarecimentos devido a uma série de limitações. Mais pesquisas precisam ser realizadas para que essa glicoproteína seja utilizada amplamente. Um ponto que permanece sem uma resposta definitiva, porém, reside na interpretação dos níveis séricos elevados de TN-C. O debate se tais níveis estariam relacionados a um mau prognóstico ou à possibilidade de melhor recuperação do paciente ainda permanece em aberto.

Apesar de Guo et al.^7^, Nozato et al.^14^, Iamanaka-Yoshida e Matsumoto^12^ e Schaefer et al.^15^ acreditarem que a TN-C poderia ser um biomarcador elegível, esses estudos ainda são inconclusivos no que diz respeito a seu papel no desfecho clínico dos sujeitos de pesquisa. São necessárias mais pesquisas para que seja possível compreender melhor o papel da TN-C no prognóstico dessas doenças.

Ante o exposto, alerta-se para a necessidade de mais pesquisas antes que a TN-C possa ser usada como biomarcador, pois se reitera a escassez de estudos. Ademais, tais estudos têm apresentado amostras pequenas, sendo, por conseguinte, difícil balizar valores de referência sem a existência de coortes validadas.
